# Respiratory Disease Classification Using NMF-Enhanced Log-Mel Spectrograms and Convolutional Recurrent Neural Networks

**DOI:** 10.3390/s26134268

**Published:** 2026-07-04

**Authors:** Bowen Han, Wei Quan, Bogdan Matuszewski, Dennis Corbett

**Affiliations:** 1 School of Engineering and Computing, University of Lancashire, Preston PR1 2HE, UK; wquan@lancashire.ac.uk (W.Q.); bmatuszewski1@lancashire.ac.uk (B.M.); 2 Department of Paediatric, East Lancashire Hospitals NHS Trust, Blackburn BB2 3HH, UK; dennis.corbett@elht.nhs.uk

**Keywords:** respiratory disease classification, non-negative matrix factorization, log-mel spectrogram, convolutional recurrent neural network, computer-assisted auscultation

## Abstract

Respiratory disease classification using lung sound recordings remains challenging due to signal interference, heterogeneous acquisition conditions, and substantial overlap among clinically related acoustic patterns. This study presents a framework for respiratory disease classification using NMF-enhanced log-mel spectrograms and deep neural classifiers. Respiratory sound recordings from two publicly available datasets were harmonized into a unified label space comprising Asthma, Bronchiectasis, Bronchiolitis, COPD, Healthy, Pneumonia and URTI. Following signal standardization and fixed-length segmentation, a non-negative matrix factorization (NMF)-based enhancement stage was applied to increase the salience of respiratory components prior to log-mel spectrogram generation. The proposed classifier was a convolutional recurrent neural network (CRNN) that combined convolutional feature extraction, bidirectional recurrent modelling, and attention-based temporal aggregation. For comparison, RDLINet, a conventional CNN, ResNet, and a YOLO-style backbone were implemented under the same preprocessing and training framework. Experimental results demonstrated that the proposed CRNN achieved the best overall performance, attaining 96.14 ± 0.50% accuracy and 94.05 ± 1.21% Macro-F1 on the unified seven-class cohort. Class-wise analysis, confusion-matrix evaluation, and output-space visualization further showed that the CRNN provided more balanced recognition across disease categories and clearer class separation than competing architectures. These findings indicate that NMF-enhanced spectro-temporal modelling combined with convolutional recurrent learning offers an effective approach for automated multi-class respiratory disease classification.

## 1. Introduction

Respiratory diseases are a major cause of illness and death worldwide, with their impact varying across regions and over time due to population ageing, environmental factors, and differences in healthcare access. Global Burden of Disease (GBD) studies have shown that chronic respiratory diseases continue to cause substantial disability and premature death, highlighting the need for earlier detection and long-term monitoring [1]. Among these conditions, chronic obstructive pulmonary disease (COPD) represents a significant share of the global respiratory disease burden and remains a leading cause of death worldwide [2]. In addition, lower respiratory infections (LRIs) continue to affect millions of people each year and are associated with considerable illness and mortality, particularly in low-resource settings [3]. These challenges emphasize the need for accessible and reliable tools that can support respiratory disease screening, diagnosis, and monitoring.

Auscultation remains one of the most widely used methods for assessing respiratory health because it is non-invasive, fast, and inexpensive. By listening to lung sounds, clinicians can identify signs of airway obstruction and other respiratory abnormalities during routine examinations. However, the interpretation of lung sounds is often subjective and can vary according to clinical experience, which has motivated the development of computer-assisted analysis methods that provide more objective and consistent assessments [4]. Efforts to standardize lung sound terminology and establish shared respiratory sound databases have further highlighted the need for reproducible computational approaches that can support consistent interpretation across clinicians and healthcare settings [5]. In addition, the growing availability of digital stethoscopes and low-cost sensing devices has accelerated research into automated respiratory disease analysis, supported by publicly available datasets containing annotated respiratory sound recordings and benchmark classification tasks [6].

A major challenge in respiratory sound analysis is that lung sounds are often mixed with heart sounds and other background noise. This problem is particularly noticeable during quiet breathing, where heart sounds can overlap with respiratory signals and make clinically important acoustic features more difficult to detect. Early studies showed that cardiac interference can significantly affect respiratory sound analysis, motivating the development of signal enhancement and noise reduction techniques before feature extraction and classification [7]. More recent research has continued to investigate methods for reducing cardiac interference, including time–frequency analysis and adaptive signal processing approaches, with the aim of improving the quality of respiratory sound representations and preserving disease-related acoustic information [8].

The proposed pipeline first applies waveform-level denoising to reduce background noise and recording artefacts, followed by non-negative matrix factorization (NMF) to enhance respiratory-dominant acoustic structures and suppress cardiac interference. The enhanced signals are then transformed into log-mel spectrograms and classified using a convolutional recurrent neural network (CRNN) with attention-based temporal aggregation. To provide a fair evaluation, RDLINet, a conventional CNN, ResNet, and a YOLO-style backbone were implemented under the same preprocessing, feature extraction, and training framework.

The main contribution of this work is the integration of signal enhancement and spectro-temporal deep learning for respiratory disease classification. By combining waveform denoising, NMF-based respiratory enhancement, log-mel spectrogram representation, and convolutional recurrent modelling, the proposed approach aims to improve the discrimination of clinically related respiratory diseases from heterogeneous auscultation recordings. Furthermore, a comprehensive comparative analysis is conducted to investigate the impact of temporal sequence modelling on disease recognition performance and class-balanced classification.

## 2. Related Work

Previous research on automated respiratory disease analysis has primarily focused on three areas: deep learning-based classification of respiratory sounds, signal enhancement techniques for reducing cardiac interference, and spectral decomposition methods for respiratory sound separation. Although substantial progress has been made in each area, reliable disease classification remains challenging due to heterogeneous recording conditions, overlapping acoustic characteristics among respiratory diseases, and the presence of non-respiratory interference. These challenges motivate the development of classification frameworks that combine signal enhancement with spectro-temporal deep learning to improve disease discrimination from respiratory sound recordings.

Deep learning has become one of the dominant approaches for automated respiratory disease classification, with most studies transforming respiratory sound recordings into time–frequency representations such as spectrograms or log-mel spectrograms. Log-mel representations have been widely adopted because they provide a compact description of spectral content while preserving important respiratory acoustic patterns [9,10]. Similar spectrogram-based Convolutional neural networks (CNNs) approaches have also been shown to be effective in general environmental sound classification tasks [11]. In respiratory sound analysis, the availability of publicly accessible datasets with annotated recordings has enabled the development and benchmarking of deep learning-based classification systems [6]. CNNs have demonstrated strong performance by learning discriminative spectral features associated with respiratory abnormalities such as wheezes and crackles [12]. However, respiratory disease signatures often evolve over time and may span multiple breathing phases, making temporal information important for accurate classification. To address this limitation, convolutional recurrent neural networks (CRNNs) have been introduced to combine convolutional feature extraction with recurrent sequence modelling, enabling the capture of both local spectral patterns and longer-term temporal dependencies [13,14]. Similar observations have been reported in sound event detection research, where recurrent modelling has been shown to improve the discrimination of acoustically similar events when temporal context is important [15].

Cardiac interference remains a major challenge in automated respiratory sound analysis because heart sounds overlap with low-frequency respiratory components and can obscure disease-related acoustic information. Early studies addressed this problem using wavelet-based techniques to suppress cardiac contamination in respiratory recordings [7]. More recently, data-adaptive decomposition methods have been investigated as an alternative approach for separating respiratory and cardiac components in complex, non-stationary signals. Among these methods, Empirical Mode Decomposition (EMD) and Variational Mode Decomposition (VMD) have been widely applied for signal denoising and component separation due to their ability to isolate oscillatory patterns with different frequency characteristics [16,17]. In respiratory sound analysis, such decomposition techniques have been used to reduce cardiac interference while preserving diagnostically relevant respiratory information prior to feature extraction and classification [8].

Although waveform-level enhancement can reduce cardiac interference and background noise, residual artefacts may still appear as overlapping structures in time–frequency representations. As a result, spectral decomposition methods are often used as an additional enhancement stage. Among these approaches, Non-negative Matrix Factorization (NMF) has attracted considerable attention for audio analysis because it decomposes spectrograms into additive components that can represent different sound sources or acoustic patterns [18]. Previous studies have shown that NMF and its extensions can support source separation, noise reduction, and signal enhancement by isolating meaningful spectral structures from complex acoustic mixtures [19,20]. These properties make NMF particularly suitable for respiratory sound analysis, where respiratory and interfering components often coexist within the same time–frequency regions.

In addition to model design, evaluation methodology plays an important role in respiratory disease classification. Respiratory sound datasets are commonly represented as segmented respiratory cycles or fixed-duration snippets, which may introduce correlations between samples originating from the same recording. Consequently, careful data partitioning is required to obtain reliable estimates of model performance. Established machine learning practice recommends appropriate training, validation, and testing procedures to support unbiased model development and evaluation [21]. Furthermore, recent studies have highlighted the risk of information leakage during preprocessing, model tuning, and performance reporting, which can lead to overly optimistic results [22]. These considerations emphasize the importance of rigorous and reproducible evaluation protocols when assessing respiratory disease classification systems. Taken together, the existing literature suggests that combining respiratory signal enhancement with spectro-temporal deep learning, while maintaining a robust evaluation framework, offers a promising approach for improving automated respiratory disease classification.

## 3. Methodology

**Figure 1:**
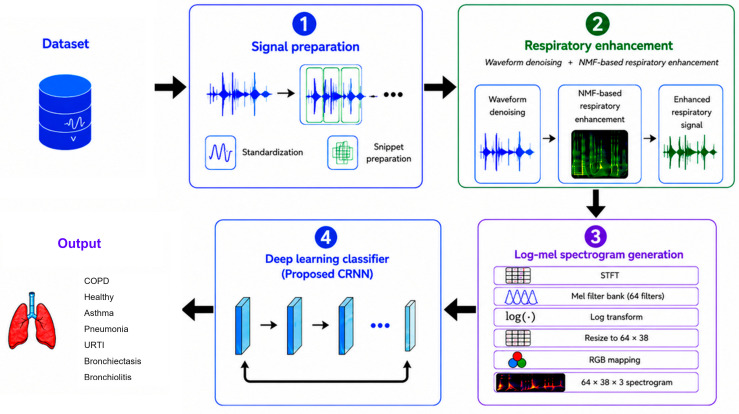
Proposed framework for respiratory disease classification.

The proposed framework performs multi-class respiratory disease classification from respiratory sound recordings through a sequential signal processing and deep learning pipeline, as illustrated in Figure 1. The workflow consists of four stages: signal preparation, respiratory sound enhancement, log-mel spectrogram generation, and disease classification. During signal preparation, recordings are converted to monaural format and resampled. Waveform-level denoising and non-negative matrix factorization (NMF) are then applied to suppress interference and enhance respiratory-dominant acoustic structures. The enhanced recordings are segmented into fixed-length snippets and transformed into log-mel spectrograms, which serve as inputs to the classification models. The primary classifier is a convolutional recurrent neural network (CRNN) designed to capture both local spectro-temporal patterns and longer-term temporal dependencies in respiratory sounds. For comparative evaluation, RDLINet, a conventional CNN, ResNet, and a YOLO-style backbone are implemented using the same input representation, training strategy, and evaluation protocol.

### 3.1. Signal Preparation

All recordings were converted to monaural format by averaging channels when necessary. To ensure consistency across datasets with different acquisition settings, all signals were resampled to a common sampling frequency of 4 kHz. This sampling rate was adopted to standardize recordings originating from multiple public databases with different native sampling frequencies while reducing computational requirements during subsequent processing and model training. A similar resampling strategy has been employed in previous respiratory disease classification studies that integrated recordings from multiple datasets into a common analysis framework [23]. Each recording was segmented into fixed-length 5 s snippets using a 50% overlap. This segmentation strategy increases the number of training samples while preserving temporal continuity between adjacent respiratory events and accommodating recordings of varying duration.

### 3.2. Respiratory Enhancement

#### 3.2.1. Waveform-Level Denoising

A waveform-level filtering stage was applied to improve signal quality by removing frequency components outside the primary respiratory sound band. Specifically, a fourth-order Butterworth bandpass filter with cut-off frequencies of 50 Hz and 1800 Hz was employed. The lower cut-off frequency was used to suppress baseline drift and other low-frequency artefacts, whereas the upper cut-off frequency was selected to reduce high-frequency noise introduced during signal acquisition. This filtering step provided a cleaner respiratory signal representation for subsequent NMF-based enhancement. Let ${\tilde{x}}_{m}\left[n\right]$ denote the standardized respiratory sound snippet. The filtered signal was obtained as

(1) $$x_{m}^{\left(d\right)}\left[n\right]=D\left\{{\tilde{x}}_{m}\left[n\right]\right\}$$

where $D\left\{\cdot \right\}$ denotes the waveform-level filtering operator. The denoised waveform is subsequently transformed into the spectrogram domain, where NMF further decomposes the signal into respiratory and interference-related components.

#### 3.2.2. Spectral Decomposition Model

**Figure 2:**
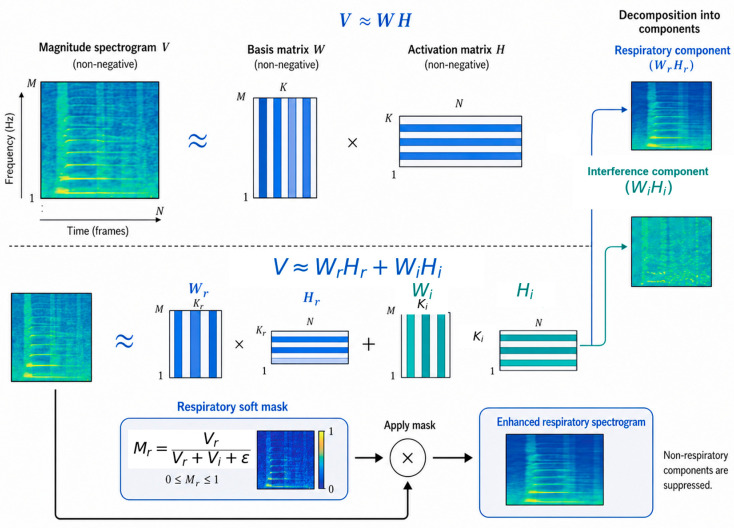
NMF-based respiratory enhancement pipeline.

The NMF based respiratory enhancement pipeline is illustrated in Figure 2. For each normalized snippet ${\tilde{x}}_{m}[n]$, a short-time Fourier transform was computed to obtain the complex spectrogram

(2) $$S_{m}(f,t)=STFT\left\{{\tilde{x}}_{m}[n]\right\}$$

where f indexes frequency bins and t indexes time frames. The corresponding magnitude spectrogram was defined as

(3) $$V_{m}(f,t)=\left|S_{m}(f,t)\right|+\epsilon$$

The NMF assumption represents the observed magnitude spectrogram as a sum of non-negative components:

(4) V≈WH

To separate respiratory and interfering structures, the dictionary matrix W was partitioned into respiratory and non-respiratory subspaces:

(5) $$W=\left[W_{r}\;W_{i}\right]$$

with corresponding activation matrices

(6) H=HrHi

The approximation therefore becomes

(7) $$V_{m}\approx W_{r}H_{r}+W_{i}H_{i}$$

Here, $W_{r}$ denotes respiratory basis vectors, $W_{c}$ denotes interfering basis vectors, and $H_{r}$, $H_{i}$ are their temporal activations.

#### 3.2.3. Objective Function and Optimization

A 512-sample analysis window, a 256-sample hop size, and a 512-point FFT were used [24]. NMF was formulated using the Kullback–Leibler divergence which is a standard objective for multiplicative-update NMF [25,26]:

(8) $$D_{KL}(V∥WH)=\sum_{f,t}\left[V_{ft}log\frac{V_{ft}}{{\left(WH\right)}_{ft}}-V_{ft}+{\left(WH\right)}_{ft}\right]$$

where $\epsilon =10^{-12}$ was used for numerical stability. Respiratory and interference-related basis dictionaries were learned separately from auxiliary recordings. The respiratory and interference dictionary ranks were set to $K_{r}=20$ and $K_{i}=10$, respectively. Since the NMF rank controls the trade-off between under-representing meaningful signal structures and modelling redundant or noise-like components [27], a larger rank was assigned to the respiratory dictionary to capture greater respiratory variability, while a smaller rank was used for the interference-related dictionary to maintain a compact interference model.

Dictionary learning was performed using 100 multiplicative-update iterations. During enhancement, the learned basis matrices were fixed and concatenated as

(9) $$W=\left[W_{r},W_{i}\right]$$

where $W_{r}$ represents the respiratory dictionary and $W_{i}$ represents the interference-related dictionary. This fixed-dictionary inference strategy follows the supervised NMF enhancement principle, where dictionaries are learned from isolated source examples and then kept fixed while activation coefficients are inferred for the mixture signal [28]. For each input recording, only the activation matrix H was updated for 60 multiplicative-update iterations:

(10) $$H\leftarrow H⊙\frac{W^{T}\left(V_{m}⊘\left(WH+\varepsilon \right)\right)}{W^{T}1+\varepsilon }$$

where ⊙ and ⊘ denote elementwise multiplication and division, respectively. The activation matrix H was initialized with random non-negative values lower bounded by $10^{-3}$. The fixed dictionaries $W_{r}\;and\;W_{i}$ were not updated during classification experiments, only H was updated for 60 multiplicative-update iterations.

To construct the basis dictionaries, the HLS-CMDS dataset was used as an auxiliary dataset for offline dictionary learning [29]. HLS-CMDS is a heart and lung sound dataset recorded from a clinical manikin using a digital stethoscope. It contains 50 isolated heart sounds, 50 isolated lung sounds, and 145 mixed heart–lung recordings, with corresponding isolated heart and lung signals available for each mixture recording. Separate respiratory and interference-related dictionaries were learned from the isolated lung and heart sound recordings, respectively, before being applied to respiratory enhancement in the proposed framework. The HLS-CMDS recordings were used exclusively for dictionary learning and were not included in the respiratory disease classification experiments.

#### 3.2.4. Respiratory Reconstruction

After the fixed activation-update stage, the activation matrix was divided into respiratory and interference-related components, denoted as $H_{r}$ and $H_{i}$. The corresponding magnitude estimates were obtained as

(11) $${\hat{V}}_{r}=W_{r}H_{r},\;\;{\hat{V}}_{i}=W_{i}H_{i}.$$

A soft respiratory mask was then computed as

(12) $$M_{r}=\frac{{\hat{V}}_{r}}{{\hat{V}}_{r}+{\hat{V}}_{i}+\epsilon },\;\;0\leq M_{r}\leq 1.$$

The mask was applied to the complex STFT of the denoised waveform, preserving the phase information for waveform reconstruction. This follows the common masking-filter strategy in audio source separation, where masks estimated from magnitude or power spectrogram models are applied to the original complex-valued spectrogram before inverse transformation [30]:

(13) $${\hat{S}}_{r}=M_{r}⊙S_{m}^{\left(d\right)}.$$

Finally, the enhanced waveform was reconstructed by inverse short-time Fourier transform:

(14) $${\overset{ˆ}{x}}_{m}^{\left(r\right)}\left[n\right]=ISTFT\left\{{\overset{ˆ}{S}}_{r}\right\}.$$

This enhanced respiratory signal was used for all subsequent feature extraction and classification stages.

**Figure 3:**
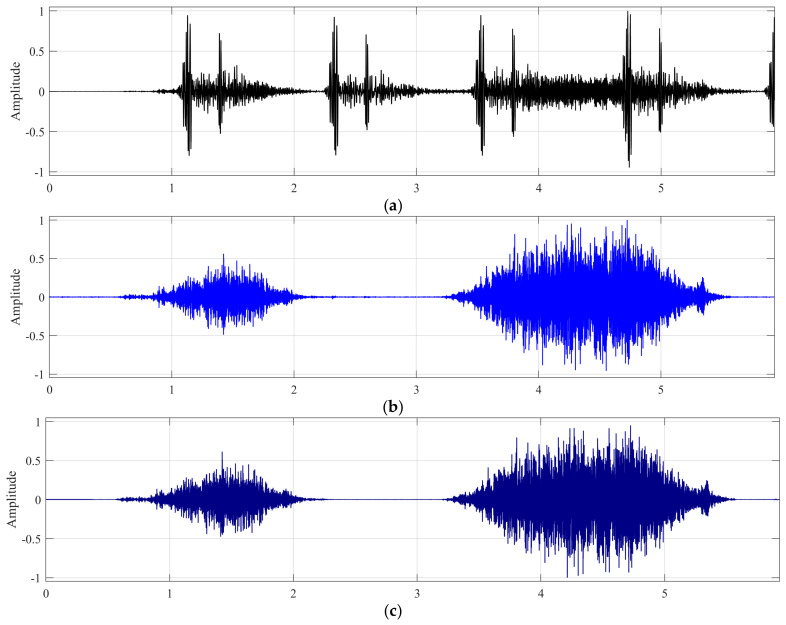
NMF-based enhancement result: (**a**) mixture signal; (**b**) ground-truth respiratory signal; (**c**) extracted respiratory signal.

Figure 3 demonstrates an example of the proposed NMF-based respiratory enhancement. Figure 3a shows the original mixture signal, which contains both respiratory and interfering components. Figure 3b presents the ground-truth respiratory signal, while Figure 3c shows the respiratory signal extracted by the proposed enhancement stage. As can be observed, the extracted respiratory signal closely resembles the ground-truth respiratory signal in terms of its temporal structure and amplitude variations. The major respiratory events are well preserved, indicating that the proposed method is capable of effectively isolating respiratory components from the mixture signal while suppressing unwanted interference. Although minor differences can be observed between the extracted and ground-truth signals, the overall signal morphology is maintained, demonstrating the ability of the proposed enhancement approach to recover clinically relevant respiratory information.

### 3.3. Data Augmentation Strategy

Data augmentation was applied to address the class imbalance problem in the datasets used in this study, where COPD constituted the majority class, while all other classes were underrepresented. To mitigate this issue, several time-domain audio data augmentation techniques were employed, including the following. It is worth mentioning that data augmentation was performed only on the training samples to prevent data leakage and ensure a fair evaluation of the model’s performance.

#### 3.3.1. Time Stretching

Time stretching modifies the temporal scale of a signal by a factor r, producing an augmented signal $x_{r}[n]$. In the present implementation, two stretching factors were adopted:

(15) $$r\in \left\{0.7,\;0.9\right\}$$

This transformation allows the classifier to observe respiratory events under slightly altered temporal dynamics, thereby reducing sensitivity to breathing rate variation.

#### 3.3.2. Pitch Shifting

Pitch shifting changes the effective frequency content of the signal by a semitone offset p. The corresponding scaling factor is

(16) $$\alpha =2^{p/12}$$

Two semitone shifts were used:

(17) $$p\in \left\{-2,\;+1\right\},$$

These perturbations were chosen to remain moderate so that disease related acoustic signatures were not excessively distorted.

#### 3.3.3. Optional Additive Noise Perturbation

In exploratory settings, an additive noise perturbation could be applied at a controlled signal-to-noise ratio. Given a clean snippet x[n], a noisy version is generated as

(18) $$x_{noise}[n]=x[n]+\sigma \eta [n]$$

where η[n] is white Gaussian noise and σ is chosen according to the desired SNR. Although this perturbation was not used in all experimental settings, the augmentation module was retained to assess robustness under future ablation studies.

#### 3.3.4. Consistent Preprocessing of Augmented Snippets

All augmented snippets underwent exactly the same preprocessing steps as the original snippets, including DC offset removal, optional baseline suppression, and amplitude normalization. This ensured consistency between original and augmented inputs and prevented distribution shifts caused by augmentation artefacts alone.

### 3.4. Log-Mel Spectrogram Generation

Each enhanced respiratory snippet ${\hat{r}}_{m}[n]$ was transformed into a log-mel spectrogram. A 1024-point analysis window, 512 sample hop size, and 64 mel filters were used. The mel-energy representation is given by

(19) $$E_{m}\left(b,\tau \right)=\sum_{f}{M_{b}(f)\left|STFT\left\{{\hat{r}}_{m}[n]\right\}\right|}^{2}$$

where b is the mel-band index and τ denotes the time frame. A logarithmic transform was applied:

(20) $$L_{m}\left(b,\tau \right)=\log\left(E_{m}\left(b,\tau \right)+\epsilon \right)$$

To ensure a fixed input size for all classifiers, the resulting map was resized to 64×38 Min–max normalization was then performed:

(21) $${\tilde{L}}_{m}=\frac{L_{m}-min(L_{m})}{max(L_{m})-min(L_{m})+\epsilon }$$

A jet colormap was applied to produce a three channel spectrogram image

(22) $$I_{m}\in R^{64\times 38\times 3}$$

This unified image representation was used across all evaluated models to ensure consistent comparison.

### 3.5. Proposed CRNN

**Figure 4:**
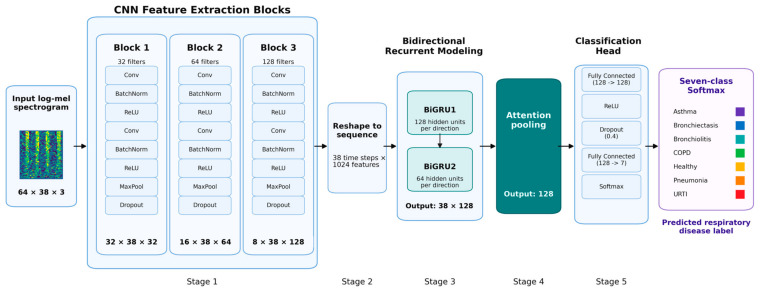
Architecture of the proposed CRNN.

The proposed classifier is a convolutional recurrent neural network (CRNN) designed to combine local convolutional spectro-temporal feature extraction with bidirectional recurrent modelling for respiratory disease classification. As shown in Figure 4, the network receives a three-channel log-mel spectrogram image of size 64 × 38 × 3, where the dimensions correspond to frequency bins, time frames, and RGB channels, respectively. The revised architecture contains six main stages: input representation, three CNN feature extraction blocks, sequence reshaping, two stacked bidirectional GRU layers, attention pooling, and a fully connected classification head. The final output is a C-class softmax.

In the convolutional front-end, local time-frequency structures are extracted through three hierarchical CNN feature extraction blocks with 32, 64, and 128 filters, respectively. Each block applies two convolutional operations, each followed by batch normalization and ReLU activation, followed by max-pooling and dropout. In this architecture, max-pooling is applied only along the frequency axis. Consequently, the temporal resolution is preserved at 38 frames throughout the convolutional front-end, while the frequency dimension is progressively compressed from 64 to 32, 16, and finally 8 frequency positions. The resulting feature-map progression is 32 × 38 ×32 after Block 1, 16 × 38 × 64 after Block 2, and 8 × 38 × 128 after Block 3.

This frequency-only pooling design is intended to reduce redundant spectral information without shortening the temporal sequence available to the recurrent layers. Pre-serving all 38 time frames is important because disease-related respiratory cues may ex-tend across multiple consecutive frames rather than being confined to isolated local regions. To make the revised feature-map progression explicit, Table 1 summarizes the main tensor sizes used by the proposed CRNN.

After the third CNN block, the feature tensor has size 8 × 38 × 128. This tensor is re-arranged into a temporal sequence by treating the 38 time frames as sequence steps and flattening the remaining frequency and channel dimensions at each time step. Therefore, each temporal step is represented by 8 × 128 = 1024 features, giving an input sequence of size 38 × 1024. Let $q_{t}$ denote the 1024-dimensional feature vector at time step t, where t = 1, …, 38. The resulting sequence is processed by two stacked bidirectional GRU layers. The first BiGRU layer uses 128 hidden units in each direction, while the second BiGRU layer uses 64 hidden units in each direction. The recurrent output therefore has size 38 × 128, corresponding to 64 forward and 64 backward features at each time step after the second bidirectional layer.

Formally, if $Q=q_{1},q_{2},\ldots ,q_{38}$ denotes the input sequence after reshaping, the hidden states of the first and second recurrent layers can be written as

(23) $$h_{t}^{\left(1\right)}={BiGRU}_{1}(q_{t},h_{t-1}^{\left(1\right)})$$

(24) $$h_{t}^{\left(2\right)}={BiGRU}_{2}(h_{t-1}^{\left(1\right)},h_{t-1}^{\left(2\right)})$$

The output sequence from the second BiGRU layer is then aggregated by an attention pooling module in order to emphasize the most informative temporal positions. For each time step, an attention score is computed and normalized across the sequence:

(25) $$e_{t}=v^{T}tanh(Wh_{t}^{\left(2\right)}+b)$$

(26) $$\alpha_{t}=\frac{exp\left(e_{t}\right)}{\sum_{j=1}^{T^{\prime }}exp(e_{j})}$$

where T = 38 is the sequence length. The final 128-dimensional embedding is obtained as the weighted sum

(27) $$z=\sum_{t=1}^{T^{\prime }}\alpha_{t}h_{t}^{\left(2\right)}$$

This mechanism enables the network to assign larger importance to respiratory frames that are more discriminative for disease recognition, while reducing the contribution of less informative temporal regions.

The aggregated 128-dimensional feature vector is finally passed through a classification head consisting of a fully connected layer from 128 to 128 units, ReLU activation, dropout with a rate of 0.4, a final fully connected layer from 128 to C units, and a softmax output. The final classification head therefore produces a softmax normalized class score vector over the target disease categories. The score associated with class c is defined as

(28) $$s_{c}=\frac{exp(o_{c})}{\sum_{j=1}^{7}exp(o_{j})}$$

where $o_{c}$ denotes the logit corresponding to class c. The final predicted disease label is obtained using the maximum a posteriori rule.

(29) $$\hat{y}=arg\;\underset{c}{\max}\;s_{c}$$

The proposed CRNN was trained using class-weighted cross-entropy loss. The AdamW optimiser was used with an initial learning rate of $3\times 10^{-4}$, a weight decay of $1\times 10^{-3}$, and a mini-batch size of 128. The model was trained for a maximum of 220 epochs, with early stopping applied when validation accuracy did not improve for 40 consecutive epochs after a minimum of 20 epochs. A ReduceLROnPlateau scheduler was used with a reduction factor of 0.5, a patience of 10 epochs, and a minimum learning rate of $1\times 10^{-6}$. Dropout was set to 0.20 after each CNN block, 0.30 after each BiGRU layer, and 0.40 in the fully connected classification head. The best checkpoint was selected according to validation accuracy. The reported CRNN results were calculated as mean ± sample standard deviation across repeated runs.

The architectural design was selected to balance representational capacity and temporal modelling efficiency. The three convolutional blocks provide local spectro-temporal feature extraction while compressing only the frequency dimension, thereby preserving a 38-step temporal sequence for recurrent modelling. The stacked bidirectional GRU layers then model forward and backward respiratory temporal context, and attention pooling aggregates the most informative frames into a compact 128-dimensional disease representation. Owing to this combination of spatial feature learning, temporal dependency modelling, and attention-based aggregation, the CRNN serves as the primary model in the proposed framework. In addition to the proposed CRNN, four comparator models were implemented under the same preprocessing, feature generation, and optimization framework to ensure a fair architectural comparison.

## 4. Experiments

### 4.1. Experimental Datasets

To rigorously evaluate the effectiveness of the proposed framework for respiratory disease classification, two respiratory sound datasets were employed in this study: the ICBHI 2017 Challenge dataset [6] and the Chest Wall Lung Sound (CWLS) dataset published by Fraiwan et al. [31].

The ICBHI 2017 Challenge dataset is one of the most widely used benchmark datasets for automated respiratory disease analysis. It contains 920 respiratory sound recordings collected from 128 subjects, including healthy individuals and patients diagnosed with asthma, bronchiectasis, bronchiolitis, chronic obstructive pulmonary disease (COPD), pneumonia, and upper respiratory tract infection (URTI). The recordings have a total duration of approximately 5.5 h and were acquired under diverse clinical conditions [6]. However, the dataset is highly imbalanced, particularly with respect to the asthma class, which contains only seven recordings. Consequently, most previous studies have focused on six-class respiratory disease classification and excluded asthma from the evaluation. To facilitate a direct comparison with existing literature and to assess the contribution of individual components of the proposed framework, a six-class respiratory disease classification experiment was first conducted using the ICBHI dataset alone.

The CWLS dataset is one of the largest publicly available respiratory sound datasets for lung disease analysis [31]. It comprises 336 lung sound recordings collected from 112 subjects, including healthy individuals and patients diagnosed with asthma, bronchiectasis, COPD, pneumonia, heart failure, and pleural effusion. In this study, only recordings belonging to the healthy, asthma, bronchiectasis, COPD, and pneumonia classes were selected, while samples associated with heart failure and pleural effusion were excluded because these disease categories are not represented in the ICBHI dataset.

To construct a more comprehensive respiratory disease cohort, the selected classes from the CWLS dataset were combined with those from the ICBHI dataset. The inclusion of the CWLS dataset substantially increased the number of asthma recordings, thereby overcoming a major limitation of the ICBHI dataset and enabling asthma to be considered as a distinct disease category. This integration facilitated the development of a seven-class respiratory disease classification framework consisting of asthma, bronchiectasis, bronchiolitis, COPD, healthy, pneumonia, and URTI. Compared with the conventional six-class benchmark commonly adopted in studies based solely on the ICBHI dataset, the proposed seven-class setting provides a more comprehensive and clinically relevant evaluation by explicitly incorporating asthma as an additional disease category.

The datasets were first partitioned at the recording level into training (80%), validation (10%), and testing (10%) subsets. All snippets extracted from the same original recording were assigned exclusively to a single subset of training, validation or testing in the following experiments. Data augmentation was then applied only to the training subset to alleviate class imbalance, while the validation and testing subsets remained unchanged to prevent data leakage and ensure an unbiased evaluation. Following augmentation, the number of 5-s snippet samples in the training portion of the ICBHI dataset increased from 6922 to 9286, while the number of samples in the training portion of the CWLS dataset increased from 1386 to 5046. Table 2 provides a summary of the training, validation, and testing subsets used for the seven-class respiratory disease study.

### 4.2. Performance Analysis

To investigate the contribution of each component of the proposed framework, an ablation study was conducted using the six-class respiratory disease classification task on the ICBHI dataset. Three configurations were evaluated: (i) a baseline CRNN using only the raw respiratory waveforms and their corresponding log-mel spectrograms; (ii) a CRNN with the waveform denoising stage applied prior to spectrogram generation; and (iii) the complete proposed framework, including waveform denoising and NMF-based respiratory enhancement. The results are presented in Table 3.

The baseline CRNN achieved an accuracy of 88.65% and a Macro-F1 score of 78.78%. After introducing the waveform denoising stage, the Macro-F1 score increased substantially to 84.98%, indicating that suppressing non-respiratory interference improved the classifier’s ability to recognize minority disease classes. The average accuracy showed an increase to 90.06% when the higher Macro-F1 score suggests a more balanced classification performance across all classes.

The best performance was achieved by the full processing pipeline, which incorporates both waveform denoising and NMF-based respiratory enhancement. This configuration obtained an accuracy of 96.84% and a Macro-F1 score of 90.23%, representing improvements of 8.19% in accuracy and 11.45 percentage points in Macro-F1 compared with the baseline model. The substantial increase in Macro-F1 demonstrates that the NMF enhancement stage further improves the salience of respiratory acoustic patterns and reduces the influence of residual interference, leading to better discrimination of minority disease categories.

The results confirm that each processing stage contributes positively to the final classification performance. While the CRNN is capable of learning meaningful representations directly from the spectrograms, the addition of waveform denoising and NMF-based enhancement produces more informative respiratory representations, resulting in a more robust and balanced multi-class classification system.

All results reported as mean ± SD were computed across 10 independent runs using different predefined random seeds. The reported standard deviation corresponds to the sample standard deviation across these repeated runs. The relatively large standard deviation of the Macro-F1 score in the denoised ablation condition reflects the higher sensitivity of this configuration to random initialisation and mini-batch sampling under the class-imbalanced dataset, particularly for the minority classes. In contrast, incorporating the NMF enhancement stage produced more stable performance across repeated runs while also improving the average Macro-F1 score.

To provide additional context for the effectiveness of the proposed framework, Table 4 presents an indicative comparison with several previously published studies that reported six-class respiratory disease classification results on the ICBHI dataset. It should be noted that this comparison is intended for reference purposes only, as the competing methods were not re-implemented or re-evaluated under the same experimental protocol in this study. Differences in data preprocessing, augmentation strategies, data partitioning, and evaluation procedures may therefore influence the reported results.

As shown in Table 4, the proposed full processing pipeline achieved an accuracy of 96.84 ± 1.43% and a Macro-F1 score of 90.23 ± 5.93%. These results are numerically comparable with, and in some cases higher than, those reported by several previously published studies on the ICBHI dataset. For example, the reported accuracy is higher than that of the multi-task learning framework [32], the parallel convolutional autoencoder approach [33], and the Deep Ensemble Neural Network [34]. The MFCC-GRU classifier [35] reported an accuracy of 95.67 ± 0.77% and a Macro-F1 score of 95.66 ± 0.79%, indicating competitive performance under its own experimental setting. However, these comparisons should be interpreted with caution because differences in data partitioning strategies, preprocessing procedures, data augmentation methods, class distributions, evaluation protocols, and performance metric calculations preclude direct benchmark comparison.

Collectively, the results suggest that the combination of waveform denoising, NMF-based respiratory enhancement and CRNN-based temporal modelling provides a competitive and effective solution for respiratory disease classification. While the comparison is only indicative, the proposed framework demonstrates performance that is at least comparable to, and in several cases exceeds previously reported results on the ICBHI dataset.

### 4.3. Seven-Class Respiratory Disease Classification

To construct a more comprehensive respiratory disease cohort, a seven-class respiratory disease classification framework consisting of asthma, bronchiectasis, bronchiolitis, COPD, healthy, pneumonia, and URTI was developed by integrating the ICBHI and CWLS datasets. Compared with the conventional six-class benchmark commonly adopted in studies based solely on the ICBHI dataset, the proposed seven-class setting provides a more comprehensive and clinically relevant evaluation by explicitly incorporating asthma as an additional disease category.

To provide a strong benchmark, four commonly used respiratory disease classification models were also implemented in this study, namely RDLINet [36], a conventional CNN [37], ResNet [38], and a YOLO-style backbone [39]. All models were trained and evaluated using the same preprocessing pipeline, input representation, data partitioning strategy, and repeated evaluation protocol to ensure a fair comparison. Table 5 summarizes the results of the seven-class respiratory disease classification task.

As shown in Table 5, the proposed CRNN achieved the best overall performance among all evaluated models, obtaining an average accuracy of 96.14 ± 0.50% and a Macro-F1 score of 94.05 ± 1.21%. Compared with the strongest baseline, the conventional CNN, the proposed model improved accuracy by 1.72% and Macro-F1 by 1.60%. These results indicate that the recurrent temporal modelling component enables the network to capture disease-related temporal dependencies that are not fully exploited by purely convolutional architectures.

The CNN achieved the second-best performance, with an accuracy of 94.42 ± 0.47% and a Macro-F1 score of 92.45 ± 1.40%, demonstrating that convolutional feature extraction alone is effective for respiratory sound classification. ResNet achieved slightly lower performance, reaching 91.92 ± 0.51% accuracy and 86.77 ± 1.81% Macro-F1, suggesting that deeper residual feature extraction does not necessarily translate into superior classification performance for this task.

The YOLO-style backbone and RDLINet produced considerably lower scores than the CRNN, CNN, and ResNet models. The YOLO-style architecture achieved 86.89 ± 0.54% accuracy and 82.89 ± 2.16% Macro-F1, while RDLINet obtained the lowest overall performance with 82.66 ± 0.80% accuracy and 75.73 ± 3.60% Macro-F1. The substantial reduction in Macro-F1 for these models indicates difficulty in handling minority disease categories and maintaining balanced performance across all classes.

Taken together, the results demonstrate that the proposed CRNN provides the most effective combination of spectro-temporal feature extraction and temporal dependency modelling for seven-class respiratory disease classification. The consistent improvements in both accuracy and Macro-F1 further suggest that the proposed framework achieves not only higher overall classification accuracy but also more balanced recognition across disease categories.

**Figure 5:**
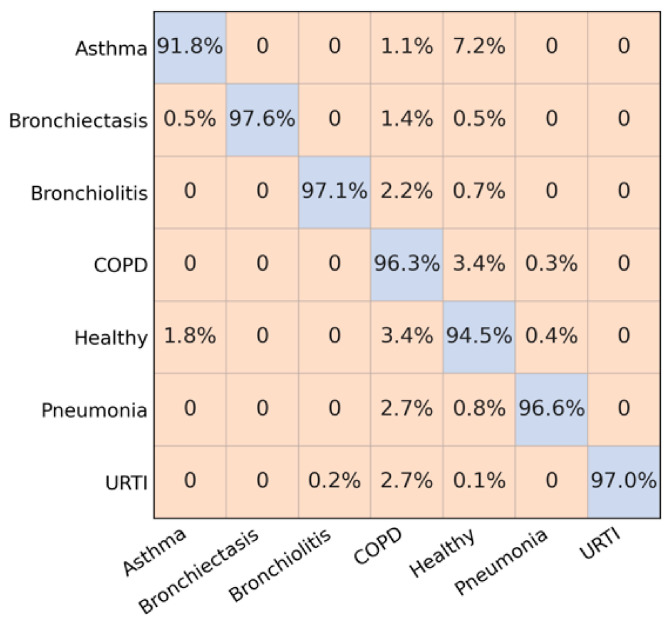
Confusion matrix of seven-class evaluation by CRNN.

**Figure 6:**
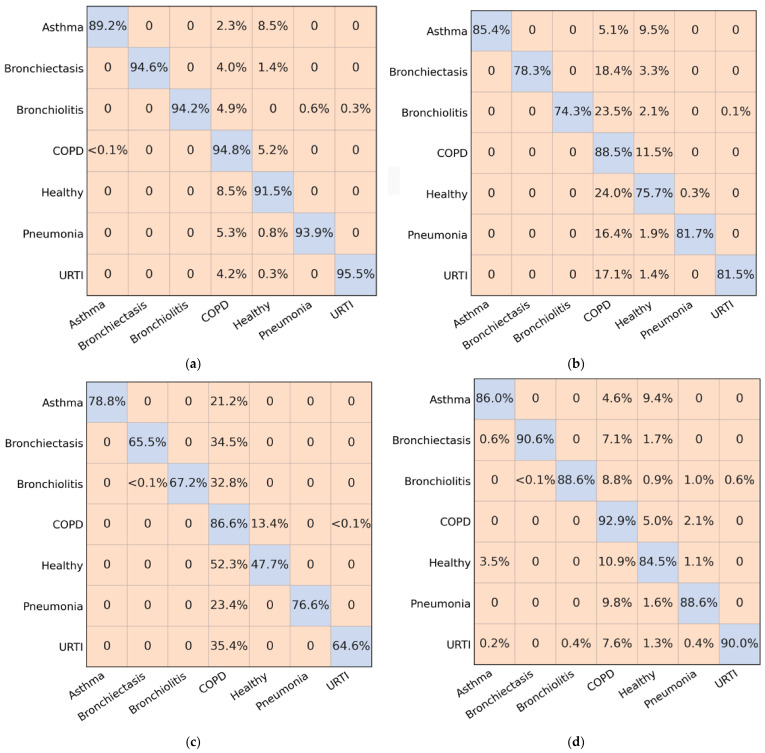
Confusion matrix of seven-class evaluation by the baseline models: (**a**) CNN; (**b**) YOLO; (**c**) RDLINet; (**d**) ResNet.

To further examine the class-wise performance of the evaluated models, confusion matrices were generated for the seven-class respiratory disease classification task. Figure 5 presents the confusion matrix of the proposed CRNN, while Figure 6 shows the corresponding confusion matrices of the benchmark models, including CNN, ResNet, YOLO-style backbone, and RDLINet.

As illustrated in Figure 5, the proposed CRNN achieves a strong diagonal distribution, indicating that the majority of samples are correctly classified across all disease categories. Only a limited number of misclassifications can be observed, suggesting that the combination of NMF-enhanced log-mel spectrograms and convolutional recurrent modelling provides effective discrimination between respiratory diseases. The relatively uniform distribution of correct predictions across classes is consistent with the high Macro-F1 score reported in Table 5, indicating balanced recognition performance for both majority and minority categories. Figure 6 reveals a greater degree of confusion among disease categories for the baseline models. Although the CNN achieves competitive performance, a larger number of off-diagonal elements can be observed compared with the CRNN, indicating increased misclassification between acoustically similar respiratory conditions.

ResNet, YOLO-style backbone, and RDLINet exhibit progressively higher levels of class overlap, which is reflected in their lower accuracy and Macro-F1 scores. In particular, these models show greater difficulty in maintaining balanced recognition across all disease categories, leading to increased confusion for minority classes.

### 4.4. Qualitative Visualization

**Figure 7:**
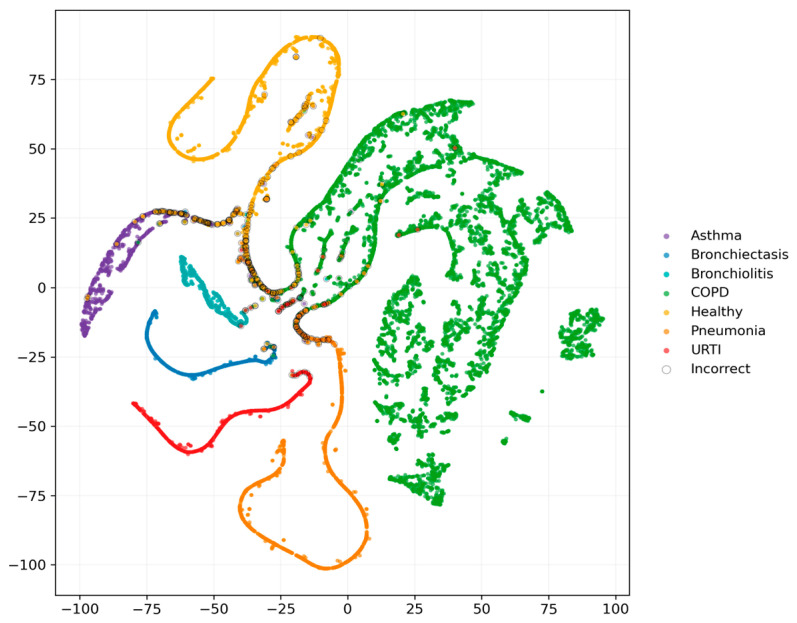
t-SNE projection of output probability vectors generated by CRNN.

**Figure 8:**
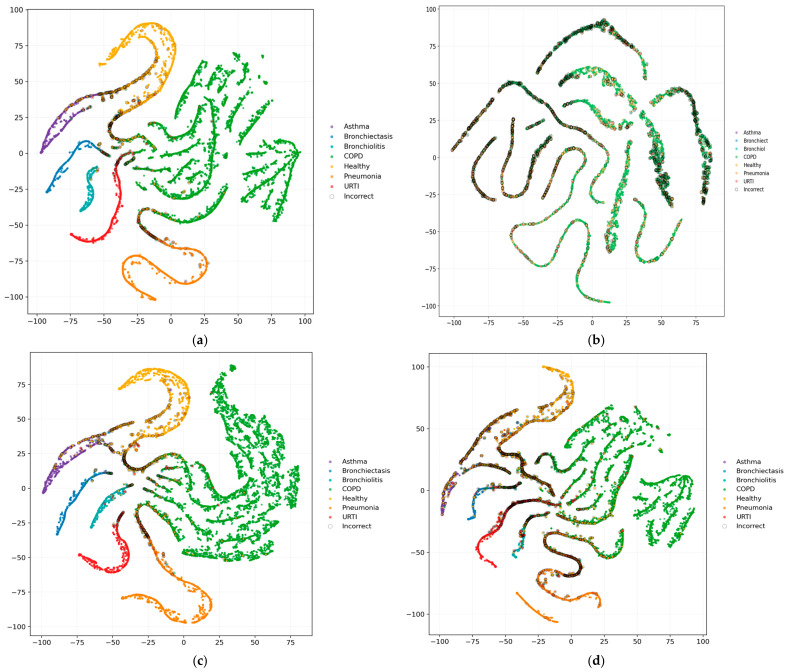
t-SNE projection of output probability vectors produced by the baseline models: (**a**) CNN; (**b**) RDLINet; (**c**) ResNet and (**d**) YOLO-style backbone.

To further investigate the discriminative characteristics of the evaluated classifiers, t-distributed stochastic neighbour embedding (t-SNE) was applied to the predicted probability vectors generated by each model under the seven-class evaluation setting. The resulting two-dimensional projections are presented in Figure 7 and Figure 8 for the proposed CRNN, conventional CNN, ResNet, YOLO-style backbone, and RDLINet. Since the visualization is derived from the final output probability distributions rather than intermediate feature representations, it should be interpreted as a qualitative illustration of class separability within the models’ decision space.

As illustrated in Figure 7 and Figure 8, all evaluated models exhibit some degree of class organization, indicating that meaningful disease-related patterns have been learned from the NMF-enhanced log-mel spectrograms. However, noticeable differences can be observed in the compactness and separation of the resulting clusters. The proposed CRNN produces the most coherent output structure, with several disease categories forming compact and well-defined groups. In particular, minority classes remain relatively distinct, suggesting that the model is able to preserve discriminative information across a broader range of respiratory conditions.

The conventional CNN generates the second most structured output distribution, which is consistent with its strong quantitative performance reported in Section 4.3. Although the CNN achieves clear cluster formation for several classes, greater overlap can be observed at the boundaries between neighbouring disease categories. ResNet demonstrates intermediate behaviour, producing more organized class groupings than the lighter architectures but exhibiting less compact separation than the CRNN. In contrast, the YOLO-style backbone and RDLINet show more fragmented cluster structures and increased inter-class mixing, particularly among the minority categories.

A large dominant manifold is visible across all projections, corresponding primarily to the majority COPD class. This behaviour reflects both the class imbalance present in the merged dataset and the substantial acoustic similarity among recordings belonging to this category. Nevertheless, the CRNN maintains clearer boundaries between this dominant class and the remaining disease categories. This observation aligns with the confusion-matrix results presented in Section 4.3, where the CRNN achieved higher class-wise recognition rates and fewer cross-class misclassifications than the competing architectures. Conversely, the more dispersed output distributions observed for the YOLO-style backbone and RDLINet are consistent with their lower Macro-F1 scores and reduced performance on minority classes.

## 5. Conclusions and Future Work

This study combined respiratory sound recordings from the ICBHI 2017 and CWLS datasets into a seven-class respiratory disease classification cohort comprising Asthma, Bronchiectasis, Bronchiolitis, COPD, Healthy, Pneumonia, and URTI. A waveform-level denoising stage and an NMF-based respiratory enhancement stage were applied before log-mel spectrogram generation to improve the salience of respiratory-dominant acoustic patterns. The enhanced spectrograms were then classified using a CRNN that integrates convolutional feature extraction, bidirectional recurrent modelling, and attention-based temporal aggregation. The experimental results indicate that the proposed CRNN consistently outperformed other models. This included traditional CNN, ResNet, YOLO-style backbone, and RDLINet. The framework achieved the highest overall classification accuracy and Macro-F1 score while ensuring a balanced recognition of different disease categories. The confusion matrix analysis demonstrated better class-wise differentiation, especially for minority classes. Visualizations using t-SNE revealed a clearer and well-separated output space. The ablation study confirmed that both waveform denoising and NMF positively impacted final classification performance. These findings show that the proposed framework effectively classifies multiple respiratory diseases and offers a good solution for computer-assisted listening and screening using lung sound recordings.

Despite the strong performance of the framework on the merged seven-class cohort, there are still several areas for further research. First, additional validation on independent external datasets is necessary to evaluate the robustness and generalisability of the proposed framework across different recording devices, acquisition environments, and patient populations. Such evaluation would provide a more comprehensive assessment of its potential for real-world clinical application. In addition, a limitation of the present study is that the datasets were partitioned at the recording level rather than the subject level. Although recordings from the same recording session were kept within a single data subset to prevent data leakage, multiple recordings from the same subject may still appear in different subsets. Future work will therefore evaluate the proposed framework using strict subject-independent data partitioning to provide a more rigorous assessment of its generalization performance. Second, future research could investigate alternative respiratory sound enhancement techniques, such as adaptive signal decomposition and deep learning-based source separation, to further improve the quality of respiratory signal representations. The effects of different spectrogram configurations and feature extraction methods should also be systematically investigated. Third, although this study focused on respiratory disease classification, incorporating uncertainty estimation and explainable artificial intelligence techniques could improve the transparency and reliability of model predictions. Visual explanation methods and confidence-aware decision systems may provide clinicians with additional diagnostic support. Finally, exploring model compression and deployment strategies would facilitate implementation of the proposed framework on resource-constrained devices, such as digital stethoscopes and edge-computing platforms. These developments would further support the translation of automated respiratory disease classification systems into clinical practice and community healthcare settings.

## Figures and Tables

**Table 1:** Summary of the CRNN feature-map dimensions.

Stage	Operation	Output Size	Role
Input	log-mel image	64 × 38 × 3	Model input
CNN Block 1	32 filters; frequency-only pooling	32 × 38 × 32	Low-level spectral features
CNN Block 2	64 filters; frequency-only pooling	16 × 38 × 64	Deeper local features
CNN Block 3	128 filters; frequency-only pooling	8 × 38 × 128	Final CNN tensor
Sequence reshape	Flatten frequency × channel per frame	38 × 1024	Temporal sequence
BiGRU 1	128 hidden units per direction	38 × 256	Bidirectional context
BiGRU 2	64 hidden units per direction	38 × 128	Compact recurrent sequence
Attention pooling	Weighted aggregation over 38 frames	128	Fixed embedding
Classification head	FC 128 -> 128; ReLU; dropout 0.4; FC 128 -> 7; softmax	7-class softmax	Seven-class disease output

**Table 2:** Summary of the training, validation and testing subsets used for seven-class study.

Split	Recording	Snippets Before Augmentation	Snippets After Augmentation	Classes
Training	936	8308	14,332	7
Validation	117	985	985	7
Test	117	1003	1003	7

**Table 3:** Individual component assessment of the proposed framework.

Assessment Type	Accuracy (%)	Macro-F1 (%)
Full processing pipeline	96.84 ± 1.43	90.23 ± 5.93
Denoised waveform and spectrogram	90.06 ± 3.72	84.98 ± 22.0
Raw waveform and spectrogram	88.65 ± 0.83	78.78 ± 7.06

**Table 4:** Indicative comparison with previously published six-class respiratory disease classification studies using ICBHI dataset.

Methods	Accuracy (%)	Macro-F1 (%)
Multi-task learning [32]	91%	89%
Parallel convolutional autoencoder [33]	94.73%	-
Deep Ensemble Neural Network [34]	95.51%	-
MFCC-GRU Classifier [35]	95.67 ± 0.77%	95.66 ± 0.79%

**Table 5:** Summary of the seven-class evaluation.

Model	Accuracy (%)	Macro-F1 (%)
CRNN	96.14 ± 0.50	94.05 ± 1.21
CNN	94.42 ± 0.47	92.45 ± 1.40
ResNet	91.92 ± 0.51	86.77 ± 1.81
YOLO-style	86.89 ± 0.54	82.89 ± 2.16
RDLINet	82.66 ± 0.80	75.73 ± 3.60

## Data Availability

The datasets analyzed in this study are publicly available from the repositories and publications cited in the manuscript. The respiratory disease classification experiments used the ICBHI 2017 Respiratory Sound Database and the chest wall lung sound dataset. The HLS-CMDS dataset was used only for auxiliary NMF dictionary learning. No new patient recordings were collected for this study. Further details of data processing are available from the corresponding author upon reasonable request.
